# Foreground Scattering Elimination by Inverse Lock-in-Like Spatial Modulation

**DOI:** 10.3390/vision4030037

**Published:** 2020-08-13

**Authors:** Yueyu Lin, Sune Svanberg

**Affiliations:** 1 National Center for International Research on Green Optoelectronics, South China Normal University, Guangzhou 510006, China; yueyu.lin@coer-scnu.org; 2 Guangdong Provincial Key Laboratory of Optical Information Materials and Technology & Center for Optical and Electromagnetic Research, South China Academy of Advanced Optoelectronics, South China Normal University, Guangzhou 510006, China; 3 Department of Physics, Lund University, P.O. Box 118, SE-221 00 Lund, Sweden

**Keywords:** vision, scattering, modulation, lock-in techniques, background subtraction, Gestalt psychology

## Abstract

We describe a simple approach to enhance vision, which is impaired by close range obscuring and/or scattering structures. Such structures may be found on a dirty windscreen of a car, or by tree branches blocking the vision of objects behind. The main idea is to spatially modulate the obscuration, either by periodically moving the detector/eye or by letting the obscuration modulate itself, such as branches swinging in the wind. The approach has similarities to electronic lock-in techniques, where the feature of interest is modulated to enable it to be isolated from the strong perturbing background, but now, we modulate the background instead to isolate the static feature of interest. Thus, the approach can be denoted as “inverse lock-in-like spatial modulation”. We also apply a new digital imaging processing technique based on a combination of the Interframe Difference and Gaussian Mixture models for digital separation between the objects of interest and the background, and make connections to the Gestalt vision psychology field.

## 1. Introduction

Unimpaired vision is a very fundamental quality of life. Most visual defects can be corrected by glasses. Glare related to slant-angle specular reflections can be reduced by polarization filters. Good vision is also clearly related to adequate illumination. Normal human color vision requires object illumination by white light to activate our three types of color receptors, signaling particular colors after processing the color sensitive absorptions of the different image parts in the brain [1,2]. These receptors in the cones have low sensitivity and are, thus, inactive in low light level environments, where instead, the more sensitive rods are employed in black-and-white colorless vision. Thus, although the reflective “colors” are clearly physically equally present in low-light conditions, night vision devices employing, e.g., electro-optical image intensifiers, give black-and-white images only, actually black and green images, because of the emission characteristics of the most efficient electro-optical phosphor. However, night color vision can be obtained by separating different color bands before amplification, and then add the color coded enhanced images into a normal color image. This can be done by image processing, following, e.g., imaging color splitting by a split mirror Cassegrainian telescope, where three images are intensified simultaneously in a single unit [3]. Alternatively, rotating color-glass sectors are used in front of the image intensifier, which produces a magnified image on a white-phosphor screen, which is then observed through co-rotating color-glass sectors for flicker-less color image formation in the brain once the rotating frequency is high enough [4].

Vision can also be strongly influenced by the properties of the light transmission medium, normally the atmosphere. While there is no major molecular absorption in the spectral region of human vision (400–700 nm), scattering due to fog or smoke can certainly strongly impair vision. A remedy, frequently used in military applications, is to perform the observation at infra-red wavelengths, where Mie and Rayleigh scattering are less prominent due to the strong wavelength dependence of these processes [5,6].

A further origin of vision impairment is the scattering of a close lying, physically structured semi-transparent layer, making the contrast or isolation of remote objects very difficult. The present paper deals with this problem. We describe the phenomenon and provide a remedy based on vision physiology. We also make a connection to well-known digital imaging processing techniques used in background subtraction, and foreground removal by integral imaging.

## 2. The Problem and Its Solution

**Figure 1:**
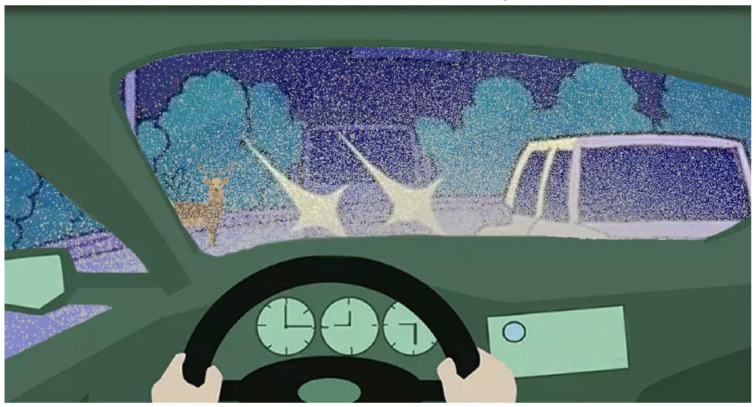
Impaired vision sometimes encountered during nighttime driving. An animal, approaching from the left, is very hard to discern.

A common imaging situation yielding strongly impaired recognition is depicted in Figure 1. The objects of interest are at a distance but are viewed through a strongly scattering window, featuring structure. Light from the close lying object completely dominates over the light derived from the distant objects of interest. An everyday situation along the lines just described is viewing through a dirty or frosty windscreen while driving, making it difficult to see the road and the objects on or adjacent to the road. The common remedy is, of course, to use an efficient screen wiper, frequently combined with spraying the screen from outside with water or an anti-frost solution. Experience shows that this does not always make the situation satisfactory, especially not in situations with counter-illumination from the sun or from the headlights from approaching vehicles. The objects of interest remain hard to discern. We illustrate this situation in Figure 1.

A further, somewhat different situation might be when the object is partly hidden by vegetation, i.e., leaves and branches, which may be moving in the wind. How could the object of interest behind be imaged clearly? Further, there may be raindrops or snowflakes falling in the line of sight to the object of interest, which impair vision. Such situations can be handled by well-known digital image processing techniques of background subtraction and integral imaging (see, e.g., [7,8,9,10,11,12,13]), but the human brain is also remarkably able to link together image elements, which are not accessible simultaneously, but only in a temporal sequence. We will discuss such situations in the present paper, while not covering cases when the object of interest is totally and permanently blocked from direct view, but where many interesting image processing approaches have recently been presented (see, e.g., [14], and references therein).

It is important to point out that we are also not considering situations where the scattering medium is more or less homogeneous, such as fog or biological tissue. Then the object of interest, frequently having sharp structures, is observed on a strong, homogenous background, making contrast minute or absent. For such situations, new and fascinating methods have recently been developed based on scattering compensation with spatial light modulators, etc. [15,16,17,18]. We are also not considering cases when the illumination of the object can be made structured, to ensure a spatial tagging, which enhances the object while reducing the non-structured background, e.g., in SLIPI (structured laser illumination planar imaging) applications [19,20]. A further way to enhance distinct distant objects over a close lying scattering medium (whether homogeneous or structured) is to simply apply far field focusing, resulting in distant objects of interest with sharp edges, which can be enhanced by digital processing [1,21,22].

The motivation to the present approach for improved vision comes from concepts of frequency and phase tagged (lock-in) detection, frequently used for retrieving weak signals residing on a strong background. The idea here is to identify the feature of interest by modulating some parameter related to the feature only, while not affecting the background. Examples of this approach are numerous in the field of spectroscopy, e.g., in wavelength modulation in diode-laser absorption spectroscopy, where the derivative of a weak but sharp absorption structure is observed by modulating the wavelength to an extent corresponding to a fraction of the feature line width (see, e.g., [23].). Another example is found in optical pumping or optical double resonance experiments [23], where the radiofrequency field inducing transitions between atomic sublevels is chopped on and off, and the minute changes in the detected light due to the action of the radio frequency are monitored selectively. The idea is to lift off and isolate the signal due to the feature of interest from the unrelated strong background. An imaging variety of such an approach could be to use modulated illumination for vision enhancement. Such illumination modulation could be taken, e.g., at nighttime driving, where the (LED) headlights of your car might be modulated in intensity (above the flicker frequency). Normal human vision would then be supported by a forward-looking video camera (possibly with a head-up display), which records synchronously and in phase with your own modulation, to enhance objects of interest to you, while direct light, or windscreen scattered light, from meeting car headlights, would be suppressed since it would be of a different modulation frequency and/or phase.

We will here focus on a different and more general approach for vision enhancement, where the enhancement occurs on the detection side only, rather than also taking the illumination side into account. One important application is when no artificial illumination occurs, e.g., in daylight applications, where the illumination obviously cannot be modulated, enabling the object of interest to be isolated from the disturbing background. The bearing principle in this paper is then to modulate instead the disturbing background, of which we can have control. It is achieved by a systematic spatial modulation by a periodic movement of the detector. If a total signal is observed and part of that signal is modulated, it can be lifted off, leaving the (frequently weak) residue isolated and clearly identifiable. Since the technique utilizes modulation, but for the opposite purpose as normally employed, the new approach could be denoted as “inverse lock-in-like spatial modulation” (ILLSPAM). A situation where spatial modulation could be implemented is the one shown in Figure 1.

## 3. Practical Implementation

The implementation of the proposed technique, using the physiology of vision and image perception, is the most straightforward application. The eye/brain system has an impressive power to “stabilize” an image, even if the detector is moving, and can easily discern the static parts of an image, while large parts of the “image” characterized by a periodic or uniform movement can be disregarded. Regarding vision psychology, there is a clear connection to the well-established *Gestalt* direction of vision apprehension (see, e.g., [24,25] and references therein). Here, the “figure–ground segregation” approach is taken, emphasizing the capability of the brain to figure out contexts, when considering the “whole” as a concept, in contrast to “the sum of the parts”; a holistic situation pertains. We, in the present treatment, more focus on the parallels to electronic signal enhancement, making the connection to electronic lock-in isolation of the feature of interest. We put our approach in perspective to all the recent technological developments in vision enhancement in environments impaired by scattering.

**Figure 2:**
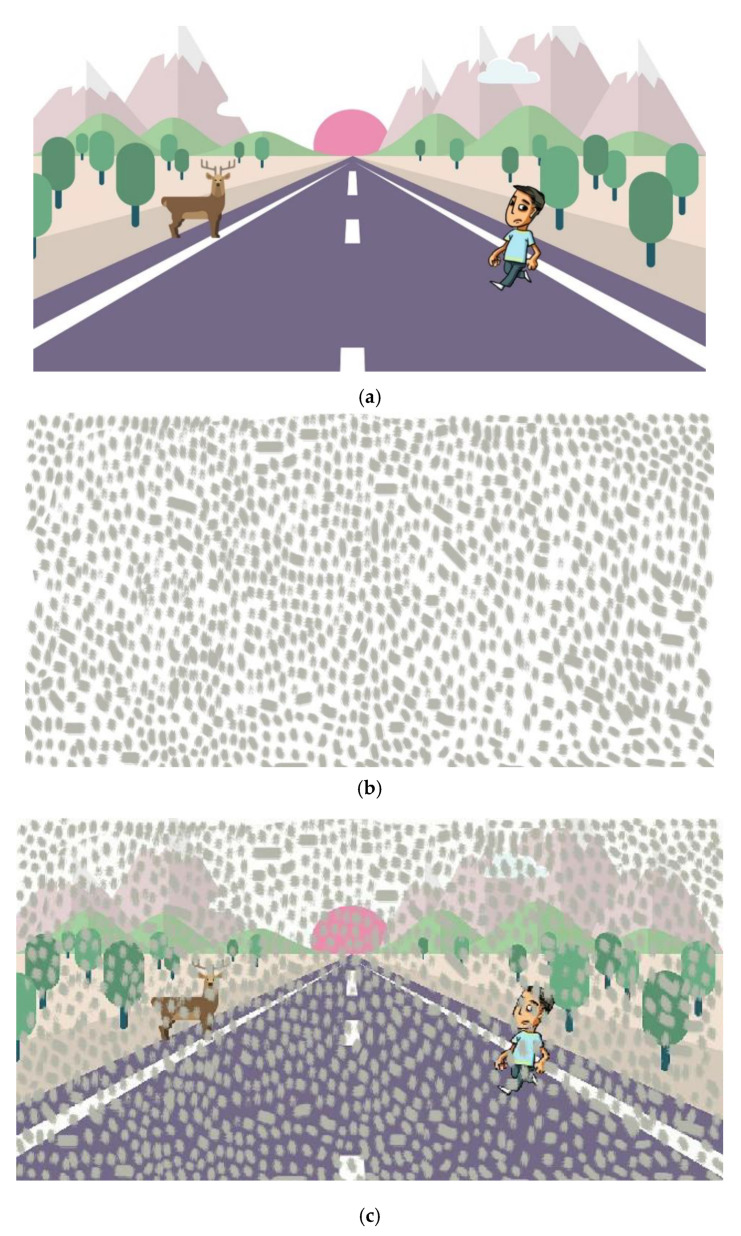
Illustration of how impaired vision sometimes encountered during car driving can be improved by a simple spatial modulation approach, utilizing the unprecedented image processing capability of the human brain. (**a**) The scene we want to observe without any image blurring; (**b**) Foreground image blurring, due to, e.g., dirt on the windscreen; (**c**) The resulting blurred image obtained as a result of the superposition of Figure 2a,b. Video S1 in the Supplementary Materials shows how Figure 2b is periodically translated up and down in front of Figure 2a, simulating a periodic movement of the observers head.

An illustrative example, which is quite useful for drivers, is illustrated in Figure 2. If surprised with a sudden situation with foreground scattering, due to the headlights of a meeting car, as illustrated in Figure 1, or the impact of strong, counter sunlight, such as in Figure 2, vision is strongly impaired. For the latter case, the resulting image Figure 2c consists of the background scene of interest, as shown in Figure 2a, and the close-by foreground scene shown in Figure 2b added scattering dirt on the windscreen. The suggested method to isolate the two scenes for obtaining a better vision of the scene in Figure 2a is to move the head a few centimeters up and down periodically and concentrate on the nonmoving parts of the scene observed. This is illustrated in the movie, connected to Figure 2c, where the foreground scattering image, Figure 2b, is moved periodically up and down to simulate the car driving situation (Supplementary Materials, Video S1). We can clearly see how the physiological vision approach works very well to increase the apprehension of the road situation, including the pedestrian and the animal.

Likewise, the eye/brain can easily see and “lock” on a building, partly blocked by, e.g., close-distance tree branches, moving in the wind and exposing different parts of the building at different times, as further discussed below.

The simple physiological approach proposed above brings to mind a further, somewhat trivial trick, well known to myopic persons, who at nighttime from the bed would see a completely blurred distant LED-based clock, which becomes crystal clear, if observed through the small opening between the thumb and two fingers brought tightly together at their tips (central-ray imaging)!

Many digital image processing approaches of removing obscuring foreground or removal of uninteresting background have been developed (see, e.g., [7,8,9,10,11,12,13]), and we will put the new physiological approach into perspective by showing an illustrative example, where a standard smartphone is utilized. The approach can be readily applied to the case of active spatial modulation of a close range partially obstructing object (say, a dirty windscreen) when the camera or an attached optical arrangement is periodically spatially translated. Cases of passive, close or far range modulation can also be considered when, e.g., vegetation, rain, or snow in motion impairs the visualization of a distant object, e.g., a building.

**Figure 3:**
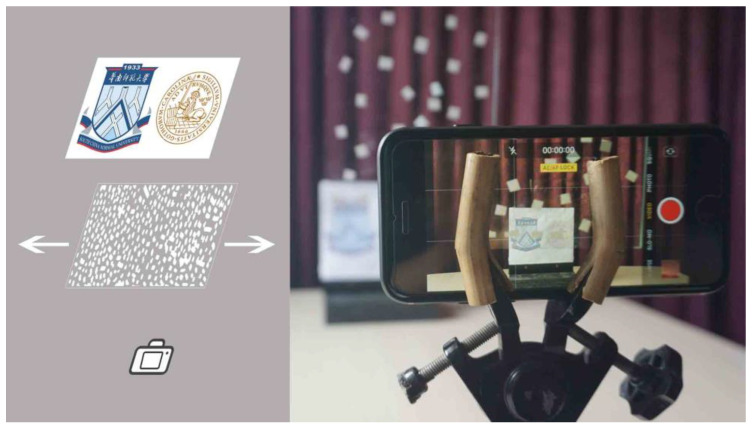
The laboratory setup with the object, the obscuring foreground screen and the smartphone video camera. (**left**) schematic setup, and (**right**) a photograph of the setup.

We will now put the physiological vision approach in relation to digital image processing and will present a method to isolate the objects of interest. Figure 3 illustrates a laboratory set up, where a fixed smartphone camera was subjected to a transparent screen, 0.5 m from the camera, which was cluttered with an irregular pattern of scattering grey spots, and which were in periodic motion. An object of interest (the logos of the participating universities) was placed at a distance of 1 m, as seen in the background of Figure 3. Images were recorded with successive displacements of the disturbing cluttering screen.

**Figure 4:**
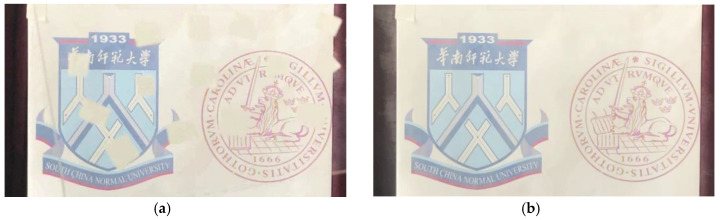
Individual frame (**a**) and a digitally processed image (**b**). Video S2 in the Supplementary Material shows a short sequence, with individual partly obscured frames to the left.

Figure 4a shows an individual frame, where parts of the object of interest were blocked, while the right-hand part shows a processed image, which was mostly free of obstruction. A short video sequence is displayed in Video S2 in the Supplementary Materials, with individual frames occurring to the left, while the video sequence, processed as indicated above, is playing to the right in the figure, without obstruction. We note that the “modulation frequency”, i.e., the rate of foreground movement should be substantially higher than that of the scene recorded to achieve an optimal processed image impression for the case that the image parts of interest would be moving. Then our procedure was performed with the reconstructed image calculated as a “sliding average”, and the objects of interest appeared in a somewhat delayed film.

The approach we used in our demonstration, shown in Figure 4, is based on two already known methods. The Interframe Difference Method [11] is commonly used in motion detection, while it is not sensitive to targets, which are moving slowly. However, the Interframe Difference Method is simple and has good stability for light variations. The Gaussian Mixture Model (GMM) [12] builds the background by using M models, which come from the intensity of pixels. It also cannot detect targets with slow speed, because it builds the background by prehistory. GMM collects the intensity of a particular pixel value over a period of time for modeling. If the target moves slowly, it can wrongly be classified as a non-interesting (background) information, because the intensity of the pixel does not change much. At the same time, the Gaussian mixture model cannot deal with the problems arising from light changes because the background quality obtained by GMM often depends on the prehistory. We operated an interframe action based on the result obtained from GMM. It improved the sensitivity of GMM to light changes and improved the quality of background models as a feedback variable. By combining the two methods in our approach, certain advantages were obtained. We proceed as outlined below:

1. To increase the adaptability in updating of the training set, we defined a time *T* as the interval for the repeated processes of adding a new frame and discarding the oldest one. To prevent excessive computation time or possible motion, *T* should not be chosen too long. At time *t*-1, we then had the data set $\chi_{T-1}$:

(1) $$\begin{array}{c} \chi_{T-1}=\left\{x^{\left(t-1\right)},\ldots ,x^{\left(t-1-T\right)}\right\} \end{array}$$

Here, $x^{\left(t-1\right)}\;$ is the current pixel value at time *t* − 1. The probability of observing the current pixel value p(x) is:

(2) $$\begin{array}{c} p\left(x\right)=\overset{M}{\underset{m=1}{\sum }}\omega_{m}\cdot \eta \left(x,\;\mu_{m},\sigma_{m}{}^{2}\right) \end{array}$$

Here, the $\omega_{m}$, values, which are positive and estimate the mixing weights, add up to one at time *t*.$\;\mu_{m}$ is the mean value of the *i*-th (1≤i≤m) Gaussian in the mixture, and $\;\sigma_{m}{}^{2}$ is the estimate of the variance that describes the Gaussian models at time *t*. η is a Gaussian probability density function:

(3) $$\begin{array}{c} \eta \left(x,\mu ,\sigma^{2}\right)=\frac{1}{{\left(2\pi \sigma^{2}\right)}^{\frac{1}{2}}}e^{-\frac{{\left(x-\mu \right)}^{2}}{2\sigma^{2}}} \end{array}$$

2. In every time period, *T*, foreground and background pixels were present at the same time. Considering the background target as always stable and similar, we ranked all the Gaussian models according to the estimated mixing weights $\omega_{m}$. So we could approximate the background model from the first B largest models (i.e., those having the largest mixing weights). The probability of observing the current background pixel value p(X|BG) should be:

(4) $$\begin{array}{c} p\left(X|BG\right)=\overset{B}{\underset{m=1}{\sum }}\omega_{m}\cdot \eta \left(x;\mu_{m},\sigma_{m}{}^{2}\right) \end{array}$$

3. When a new frame comes (at time *t*), we did the following calculation for all the pixels in this frame. We calculated the distance between the pixel value *x* and the mean value of all Gaussian distributions in the mixed Gaussian model. If one of the distances was less than 2.5 times its standard deviation, we could say the pixel value *x* was defined as a background pixel belonging to the *i*-th (1≤i≤m ) Gaussian model. By doing this, we could figure out the background (static parts) and foreground (moving objects) in the frame. After calculation, the frame pixels could be divided into foreground and background pixels according to the following relation:

(5) $$\begin{array}{c} f_{c}\left(x,\;y\right)=\{\begin{array}{c} B\left(x,\;y\right),\;if\;\left|f_{c}\left(x,\;y\right)-\mu_{m,t}\right|\leq 2.5\sigma_{m,t}\\ F\left(x,y\right),otherwise \end{array}, \end{array}$$

Here, $f_{c}\left(x,\;y\right)$, B(x, y) and F(x, y) are the current frame, background frame parts (static parts), and the current foreground frame parts (moving objects).

4. After step 3, we built a temporary background image BG(x, y)  based on the background *B_n−1_* corresponding to the previous frame. The pixels in *B_n−1_* which locate in the area of B(x, y) in function 5 were updated by using the pixel value which was in the same location $f_{c}\left(x,\;y\right)$. The other pixels in the area of F(x,y) in function 5 remained the same.

5. We converted the current frame $f_{c}\left(x,\;y\right)$ and temporary background image BG(x, y) at time *t* to gray images $f_{cg}\left(x,\;y\right)\;and\;BG_{g}\left(x,\;y\right).\;$. Then we did the subtraction with the two pixel values of the corresponding position. If the distance between the pixel in $f_{cg}\left(x,\;y\right)$ and the corresponding pixel in $BG_{g}\left(x,\;y\right)$ was smaller than an adaptive threshold *H*, this meant that the current pixel could be consider as a background pixel. The gray frame could be divided into gray foreground and gray background according to the following relation:

(6) $$\begin{array}{c} f_{cg}\left(x,\;y\right)=\{\begin{array}{c} B_{g}\left(x,\;y\right),\;if\;\left|f_{cg}\left(x,\;y\right)-BG_{g}\left(x,\;y\right)\right|\leq H\\ F_{g}\;\left(x,y\right),otherwise \end{array}, \end{array}$$

6. After step 5, we could reconstruct the new background *B_n_* at time t based on the background *B_n−1_* corresponding to the previous frame. The new background *B_n_* could be obtained as follows: Based on *B_n−1_*, update the pixels which locate in the area of $B_{g}\left(x,y\right)$ in function 6 by using the pixel values which are in the same location in the current frame $f_{c}\left(x,\;y\right)$. The other pixels which locate in the area of $F_{g}\left(x,y\right)$ remain the same.

7. When a new frame arrives, loop steps 1 through 6 to update the background.

**Figure 5:**
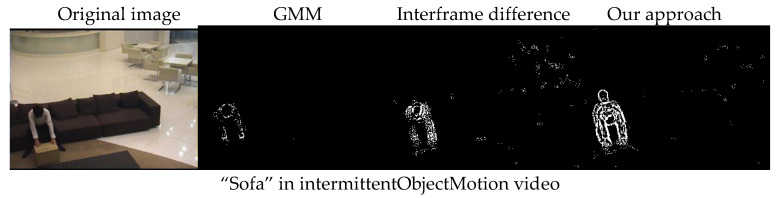

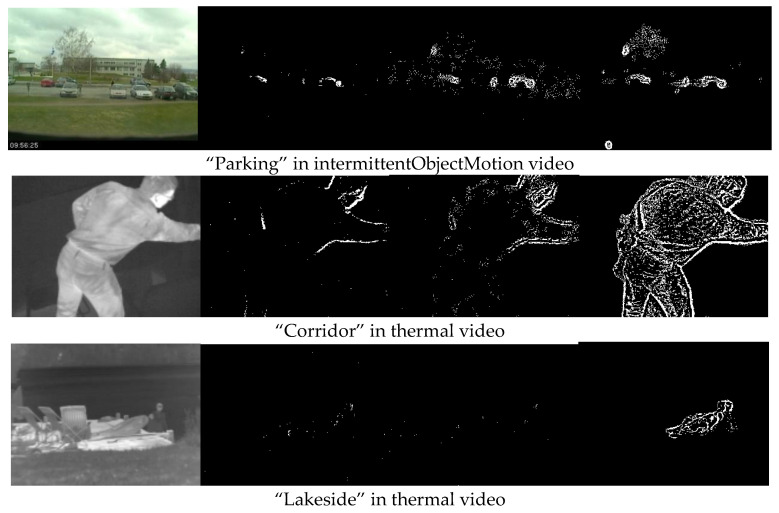
Comparison of benchmark videos between our approach and other approaches. From left to right for each scene, we present the original image, and the results for the foreground (the moving part) obtained with the Gaussian Mixture Model (GMM), the Interframe Difference Model and our new approach.

Based on the result of the Gaussian mixture model (GMM), we performed a subtraction with the corresponding frame, which means that we combined these two methods. To evaluate the results of our approach, we used the CDnet2014 data [13] to test it. We used the “intermittentObjectMotion” and “thermal video” data files available at the site. We compared our method with the Gaussian Mixture Model (GMM) [12] and the Interframe Difference Method [11]. The data set also has an official test code that specifically tests the effectiveness of different methods. However, the test code only tests the moving parts to evaluate the results of background reconstruction. Now, instead of eliminating the moving parts of the image, as done in Figure 4, we focused on the moving parts. The results regarding the identification of the moving parts (the foreground) are shown in Figure 5.

We note that a better image comprehension was obtained with our new method. However, we also applied more objective general foreground detection evaluation indices to compare the performance of our approach with two other approaches. The indices *Recall, Precision, PWC,* and *F-measure* were chosen, with the following definitions:

(7) $$\begin{array}{c} Recall=\frac{TP}{TP+FN} \end{array}$$

(8) $$\begin{array}{c} Precision=\frac{TP}{TP+FP} \end{array}$$

(9) $$\begin{array}{c} PWC=100\;*\frac{\;FN+FP}{TP+FN+FP+TN} \end{array}$$

(10) $$\begin{array}{c} F-measure=2\;*\frac{\;Precision\;*\;Recall}{Precision+Recall\;} \end{array}$$

**Figure 6:**
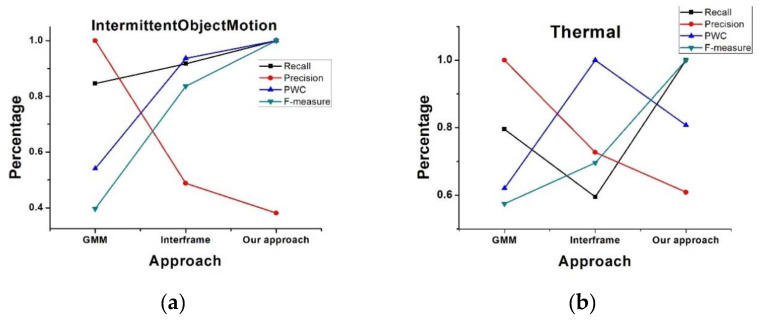
Comparison of evaluation indices of various approaches. (**a**) Performances of different approaches in intermittentObjectMotion video. (**b**) Performances of different approaches in thermal video.

Here, *TP, TN, FP,* and *FN* denote true positive rate, true negative rate, false positive rate, and false negative rate regarding the pixel identification. After running the test code, the indices were generated automatically. Each type of sequence contained different kinds of categories, and the average indices were saved in a text file. The average results are shown in the form of a line chart in Figure 6.

From 6a, we can see the *Recall, PWC,* and *F-measure* curves were better than for the other two approaches applied to the intermittent ObjectMotion videos. Although *PWC* of our approach was not the best in the thermal videos (right), the *Recall* and *F-measure* values were still good. We can also see that *Precision* of our approach was the worst in both two types of videos, which was due to our sensitivity to light change. In a next step, we will focus on the ability to handle light changes to achieve higher *Precision* values.

## 4. Discussion

We note that the aspect primarily utilized in the present approach to foreground scattering elimination was that the object of interest does not move, or at least does not move in concord with the imposed modulation. We employed modulation (by, e.g., moving the eyes/head periodically slightly up and down) at a rate allowing the object of interest to be quasi static over a period when the foreground blurring pattern moved periodically. We considered the parallel to normal lock-in (frequency- and phase-tagged) detection. In this case, the modulation must be fast enough to move away from the strongest influence of the 1/f noise in the non-interesting, overwhelming signal contributions that we wanted to discriminate against. Here, an increased spatial modulation frequency allowed us instead to mark the background clearly so that it could be discerned against the desired, weaker signal. We achieved the improved vision by using the image processing capability of the human brain, as shown in Figure 2, or by computerized image processing, as illustrated in Figure 4.

Instead of moving the detector, the foreground scattering object may instead be moved. Again, the physiological image processing of the brain could be used similarly, as illustrated in Figure 2. Clearly, here, digital image processing of the recordings of a fixed camera could also be used to eliminate the disturbing, partially obstructing close range perturbation, as illustrated in Figure 4. Incidentally, we note that a rotating round window is sometimes installed on the bridge of ships—but now for the purpose of screen wiping without moving the wiper, but rather the window. We note that even if there were no wipers, the present method would allow better vision of distant objects.

We introduced a new method, combining the established Interframe Difference and Gaussian Mixture Models to digitally separate moving and static parts of a video sequence, and demonstrated certain advantages, while there is still room for improvements, by appropriate handling of intensity variations.

We noticed that in the physiological imaging process, the brain over some time “remembers” the parts of the scene, which were non-obscured at a certain time, and is able to add to that later observed parts, which were subsequently uncovered, and finally, to synthesize a full image of the object, at any time partially obscured. This relates to the Gestalt direction of vision psychology [24,25]. This field, with origins back over 100 years [26], continues to attract much attention (see, e.g., [27,28], and analyzes the fascinating capability of human vision to ensure that “the perceptual whole is more than the sum of the parts”.

Customary imaging techniques used in video conferencing could apply, where only the moving parts of the frame (e.g., the mouth of a speaking person) are updated at video rates, while the “static” features, such as the conference room, remain at a relatively slow updating rate, thus massively saving transmission bandwidth. In the cases that we discuss, we instead eliminated the moving parts and focused on the “semi-static parts”, which constituted our object to be visualized. Again, the analog to the lock-in concept comes to mind; however, now ignoring the modulated part and focusing on the “static background”–making further meaning to the suggested acronym “inverse lock-in-like spatial modulation, ILLSPAM”. We note that the concept of modulating an accessible background instead of the feature of interest, when inaccessible for modulation, may have important applications also outside the field of vision.

## Supplementary Materials

The following are available online at https://www.mdpi.com/2411-5150/4/3/37/s1, Video S1: Figure 2 animation; Video S2: Figure 4 animation.
